# Challenges and Opportunities to Improve Cervical Cancer Screening Rates in US Health Centers through Patient-Centered Medical Home Transformation

**DOI:** 10.1155/2015/182073

**Published:** 2015-01-21

**Authors:** Olga Moshkovich, Lydie Lebrun-Harris, Laura Makaroff, Preeta Chidambaran, Michelle Chung, Alek Sripipatana, Sue C. Lin

**Affiliations:** ^1^Department of Epidemiology and Biostatistics, University of Maryland, College Park, MD 20742, USA; ^2^Office of Research and Evaluation, Office of Planning, Analysis and Evaluation, Health Resources and Services Administration, U.S. Department of Health and Human Services, 5600 Fishers Lane, Rockville, MD 20857, USA; ^3^Office of Quality and Data, Bureau of Primary Health Care, Health Resources and Services Administration, U.S. Department of Health and Human Services, 5600 Fishers Lane, Rockville, MD 20857, USA

## Abstract

Over the last 50 years, the incidence of cervical cancer has dramatically decreased. However, health disparities in cervical cancer screening (CCS) persist for women from racial and ethnic minorities and those residing in rural and poor communities. For more than 45 years, federally funded health centers (HCs) have been providing comprehensive, culturally competent, and quality primary health care services to medically underserved communities and vulnerable populations. To enhance the quality of care and to ensure more women served at HCs are screened for cervical cancer, over eight HCs received funding to support patient-centered medical home (PCMH) transformation with goals to increase CCS rates. The study conducted a qualitative analysis using Atlas.ti software to describe the barriers and challenges to CCS and PCMH transformation, to identify potential solutions and opportunities, and to examine patterns in barriers and solutions proposed by HCs. Interrater reliability was assessed using Cohen's Kappa. The findings indicated that HCs more frequently described patient-level barriers to CCS, including demographic, cultural, and health belief/behavior factors. System-level barriers were the next commonly cited, particularly failure to use the full capability of electronic medical records (EMRs) and problems coordinating with external labs or providers. Provider-level barriers were least frequently cited.

## 1. Introduction

Over the last 50 years, the incidence of cervical cancer has dramatically decreased as a result of available screening tests for early detection and intervention [1, 2]. However, health disparities persist in cervical cancer incidence and death rates for women from racial and ethnic minorities and those residing in rural and poor communities [3]. Namely, African American women are more likely to be diagnosed with cervical cancer while Hispanic/Latina women have the highest cervical cancer incidence rate [4]. Geographically, white women living in Appalachia have a much higher risk for developing cervical cancer than other white women [5]. Much of these disparities can be attributed to lack of screening, health insurance coverage, and access to health care [6]. Cervical cancer screening (CCS) interventions have been developed for minority populations; however, the efficacy of these interventions has been mixed [7–9].

To improve the overall quality of health care service delivery, the Patient Protection and Affordable Care Act of 2010 contained several provisions, which targeted the establishment and promotion of the patient-centered medical home (PCMH) model [10–12]. The PCMH model is a care delivery model with five primary attributes of comprehensive care, patient centeredness, coordinated care, accessible services, and quality and safety [13]. The enhanced access, planning, management, and monitoring of care within the PCMH model are integral for ensuring that patients receive important preventive services, including CCS [14–17]. Access to PCMH will address the health care disparities experience by underserved populations [18].

In 2012, federally funded health centers (HCs) served 21 million patients or nearly 1 in 15 Americans [19]. Among the patient population, 36% of patients were uninsured, 93% were below 200% of federal poverty level, and 62% were from racial and/or ethnic minority populations and on average 58% of female patients (aged 24 to 64 years) have received CCS [20]. In 2011, the HCs' Uniform Data System (UDS) annual clinical performance report indicated that on average 58% of female patients (aged 24 to 64 years) have been screened for cervical cancer in the past 3 years based upon the 2003 United States Preventive Services Task Force (USPSTF) recommendations on screening for cervical cancer [20, 21]. The 2009 Health Center Patient Survey indicated 85% CCS rate from patient self-report as compared to 83% CCS rate from self-report data in the National Health Interview Survey [22–24]. Efforts are underway to understand the gap between self-reported and clinically reported CCS rates and to foster strategies that will bridge the gap.

Given that adoption of the PCMH model has shown promise in increasing preventive services among underserved populations, HCs undergoing PCMH transformation have the potential to increase rates of CCS. To enhance the quality of care at HCs and to ensure more women from underserved communities are screened for cervical cancer, the Health Resources and Services Administration (HRSA) provided funding in 2012 to assist over eight hundred HCs with PCMH transformation and CCS rates improvements. In this study, we conducted a qualitative analysis with project narratives submitted by the HCs aimed at the following: (1) describe the barriers and challenges to conducting CCS in HRSA-supported HCs undergoing PCMH transformation, (2) describe the potential solutions and opportunities to improve screening rates, and (3) examine whether there were any patterns in barriers or solutions by HC characteristics, including PCMH recognition, screening rate, and proportion of uninsured patients. To our knowledge, this is the first study to examine the barriers and solutions to increase CCS in HCs within the context of the PCMH model.

## 2. Materials and Methods

### 2.1. Data Sources

In September 2012, HRSA received a total of 811 applications for funding for PCMH transformation and improvements in CCS. Applicants were asked to describe the key clinical and nonclinical activities that would support PCMH transformation/recognition and improve CCS and delineate challenges that would be addressed with the funding. In addition, the applications required a work plan, including key milestones and personnel responsible for each activity. As of February 2013, when sampling for this study took place, 809 grantees had accepted the supplemental funding.

### 2.2. Sampling Method

To select a representative sample of subject HCs, we conducted a purposive sample of the HCs using a number of characteristics from the 2011 UDS. The UDS is a core dataset of annual operation and performance of HCs where the data are aggregated at the HC organizational level. We considered the following factors to ensure that the sample reflected the diversity of federally funded HCs and their patients: total patient volume, geographical region, number of full-time clinicians per 10,000 medical patients, urban/rural location, CCS rate adjusted quartile (i.e., relative ranking of Pap test rates compared with other HCs nationwide, after accounting for differences that influence clinical performance), PCMH recognition status, percentage of homeless patients, percentage of agricultural workers patients, percentage of uninsured patients, percentage of major racial/ethnic minority groups, and percentage of patients best served in a language other than English [25, 26]. Given that barriers to CCS may be culturally specific, HCs with high concentrations of specific groups were purposively sampled.

### 2.3. Coding and Analysis

We used Atlas.ti software to assist with the coding of project narratives from subject HCs. We assessed interrater reliability by calculating Cohen's Kappa for the subsample of project narratives [27]. The Kappa coefficient assessing interrater reliability was 0.68, indicating fair interrater concordance. We exported the Atlas.ti coding data to Excel in table form for classical content analysis, to compare common themes in barriers/solutions among groups of HCs. The data consisted of binary variables (0: not coded, 1: coded) for each unique code across the subject HCs. In addition, we created “code families” in Atlas.ti, where barriers or solutions with similar themes were grouped together (e.g., “offer childcare, mobile health van, extend clinic hours” were grouped into “facilitate access”). Overall trends were identified by examining the percentage of all sampled narratives coded with various barriers and solutions. In addition, differences in the frequency of codes were examined by comparing groups of HCs: PCMH recognized (*n* = 19) versus nonrecognized (*n* = 61), top quartile CCS rate (*n* = 17) versus bottom quartile (*n* = 19), and greater than 50% of patients uninsured (*n* = 20) versus less than 25% of patients uninsured (*n* = 23).

The study was approved by the Institutional Review Board at the University of Maryland, College Park.

## 3. Results and Discussion

### 3.1. Results

#### 3.1.1. Health Center and Patient Characteristics

Table 1 compares all HCs receiving PCMH funding with those included in the study sample, which demonstrates the similarities in HC and patient characteristics between the selected sample and the universe of HCs. For instance, 23.8% of the selected sample of HCs were PCMH recognized, compared with 23.5% of all supplemental funding HC organizations. In addition, 21.3% of selected sample of HCs were in the top quartile for cervical cancer screening rates, compared with 24.2% of all organizations. For proportions of uninsured patients: 23.8% of selected sample of HCs had at least 10% of patients who were uninsured, compared with 22.5% of all HCs.

#### 3.1.2. Barriers to Improving Cervical Cancer Screening Rates

Table 2 provides a summary of patient-level, provider-level, and system-level barriers. HCs more frequently described barriers related to the patient population (e.g., seeking only symptomatic care, mistrust of the medical community) or barriers related to their infrastructure (e.g., poor record keeping, coordination with external providers or laboratories, and lack of clinical staff or coordinators). Fewer HCs identified provider-level barriers, such as noncompliance or general lack of training.

#### 3.1.3. Solutions for Improving Screening Rates

Table 2 presents major themes in solutions, organized by patient-targeted solutions, provider-targeted solutions, and system-level changes. Several key themes are described in the following sections.

*(1) Patient-Level Solutions*

*Education, Promotion, or Outreach to Patient Population.* The majority (74%) of HCs in the sample planned some kind of educational or promotional program to increase the patient population's awareness of CCS need and/or their awareness of the services offered by the HC. Several HCs planned on hosting or participating in cultural events to engage their patient population. For example, four HCs planned to partner with promotoras, Hispanic community health workers, to help with these efforts:

*“The American Cancer Society has a volunteer promotoras program, involving several Spanish speaking women, and has offered to partner with [health center] by training its volunteers in cervical cancer specific information and methods for facilitating referrals for clinical testing.”*

**Figure 1:**
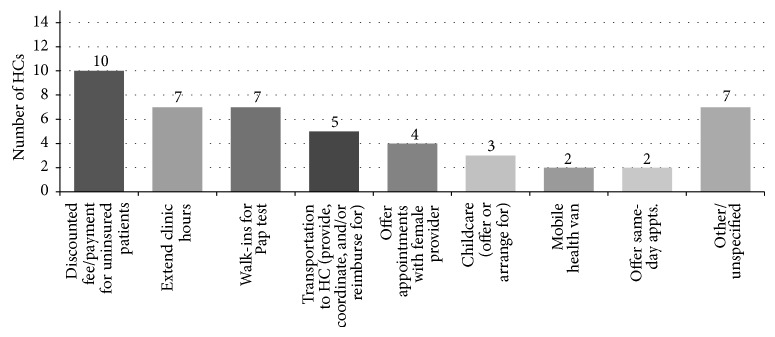
Strategies to facilitate patient access to care.

*Facilitating Access for Patients.* Approximately one-third (36%) of HCs planned to use funding in order to address patient access barriers. As shown in Figure 1, HCs aimed to address a variety of health care access issues, from financial barriers to transportation. These findings help illustrate why the PCMH model may be especially effective when serving an economically disadvantaged patient population. For instance, one HC stated that

*“Health centers are making it easier for women who cannot afford to ‘take off work' or have difficulty securing childcare meet their preventive care needs.”*

**Figure 2:**
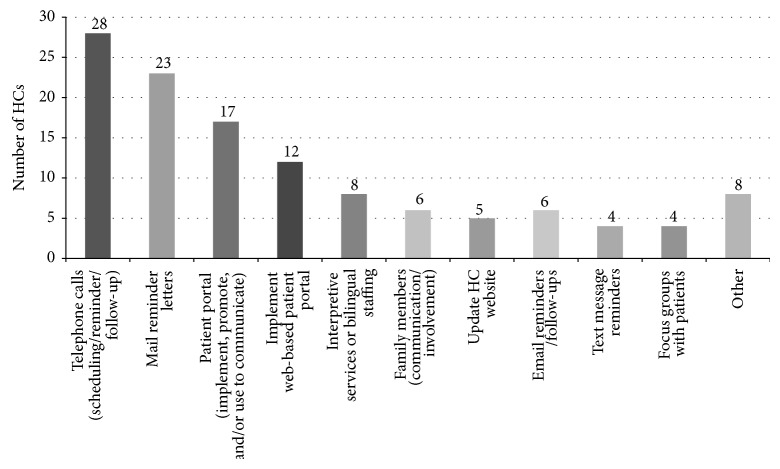
Strategies to improve communication with patient.

*Improving Communication with Patients.* The majority of HCs (66%) aimed to improve communication with patients (Figure 2). While telephone calls and mailed reminder letters were the two most popular methods for reaching patients, several HCs also planned to engage via online patient portals (either implementing a patient portal for the first time or trying to increase the number of patients enrolled).

*“A health educator will lead the development of a linguistically- and culturally-appropriate curriculum for patients on how to use the patient portal to access important health screening information such as when Pap tests are due, the importance of Pap testing, and the current screening guidelines. The health educator will partner with community advocates to implement the curriculum in the community and in the clinic to encourage women to access the patient portal and to access cervical cancer screenings at the [health center].”*

Fewer HCs proposed using email and text messages, which can be advantageous strategies to communicate with a transient patient population. Examples of other strategies to improve communication included providing a health care plan for patients, improving the telephone system, and conducting home visits to patients.

*(2) Provider-Level Solutions*

*Training and Education for HC Staff.* Although few narratives discussed provider-level barriers, more than half (55%) of HCs planned on providing some training or education for providers. Most frequently, the focus of training was on proper use of HIT. Other topics included up-to-date PCMH transformation, CCS guidelines, CCS need, cultural issues or patient communication, and clinical procedures or motivational interviewing skills.

*Other Provider-Targeted Solutions*. Examples of additional proposed methods to improve provider compliance included providing feedback on team performance or individual performance, conducting chart reviews, or offering a clinical quality bonus for providers.

*(3) System-Level Solutions*

*Hiring or Increasing Hours for Employees.* Staffing was a common way to allocate funding. While HCs strategized based on their particular need, the most frequent addition to the staff was someone who would specialize in coordination of care, or a “Patient Navigator,” who would also act to help patients navigate the health care system and help them access resources. For example,

*“With assistance and support from [health center]'s Ob/Gyn PCMH Team Leader and Chief Operating Officer, the Patient Navigator's primary function will be to guide these patients through [health center]'s health care system by addressing access issues; facilitating interaction and communication between patients and [health center] staff; and tracking interventions and outcomes.”*

*Quality Improvement (QI) Processes.* Of the 39 HCs that focused on quality improvement, a total of 16 discussed Plan-Do-Study-Act (PDSA) cycles or a similar process. A total of 10 HCs planned on conducting patient surveys. For example, one narrative described that the HC planned to

*“administer feedback/survey processes through a variety of methods including personal interviews in exam rooms, focused feedback with survey cards, SurveyMonkey, Facebook, provider/staff interviews, and so forth.”*

*Utilizing Health Information Technology (HIT)*. While almost all HCs had implemented an electronic medical record (EMR) system, the majority (79%) proposed solutions to improve their use of EMR or to increase their EMR capability. Most frequently, HCs planned to use their EMR to identify patients in need of CCS by creating a registry (61%) and/or flagging patients due for a test (36%). Other examples of improving HIT included clinical decision support tools, downloadable patient educational materials, tracking provider compliance, integrating EMR with diagnostic equipment, and documenting efforts to reach patients in the EMR.

*Protocols to Better Manage Care and Workflow Changes.* A total of 32 narratives described a protocol to be implemented for managing patient care. Examples of these solutions included bundling CCS with other services, developing a comprehensive health assessment questionnaire, and previsit planning. Similarly, several HCs planned to implement new front desk procedures, such as offering appointments with a female provider, verifying patient contact information at each patient visit, and offering CCS appointments for female patients.

#### 3.1.4. Patterns across Health Center Characteristics

*Findings by PCMH Recognition*. Solutions differed in frequency across project narratives according to PCMH recognition status. While there were few substantial differences, results suggest that HCs with PCMH recognition proposed more patient-centered strategies on how to facilitate access, with consideration of the patient population's unique cultural needs. For example, HCs with PCMH recognition status (*n* = 19) were more likely to develop or provide educational materials in other languages (26% versus 16%), utilize a mobile health care van (11% versus 0%), arrange for walk-ins for Pap tests (16% versus 7%), and utilize a community health worker (11% versus 3%), compared with HCs without PCMH recognition. In contrast, HCs that had not attained PCMH recognition were more likely to focus on telephone calls (29% versus 21%), coordination with external lab/providers (30% versus 16%), and EMR reminder tools (39% versus 26%).

*Findings by Cervical Cancer Screening Rate Quartile*. HCs in the top quartile for screening rates were more likely to plan for one-on-one patient education/intervention (47% versus 21%) and patient surveys (29% versus 11%) than HCs in the bottom quartile. Bottom quartile HCs were more likely than top quartile HCs to include education/training for providers as a solution to improve screening rates (79% versus 41%).

*Findings by Percentage of Uninsured Patients.* HCs with greater than 50% of uninsured patients were more likely to discuss specific barriers in the narratives and more likely to identify patient-level barriers, particularly cultural-related barriers, compared with HCs with less than 25% uninsured patients. HCs with a large proportion of uninsured patients were more likely to focus on facilitating access and conducting health promotion or outreach activities within the community, as well as one-on-one patient education or intervention. While both groups of HCs were equally likely to focus on patient communication, HCs serving largely uninsured patient populations were less likely to rely on traditional methods like mailed letters and telephone calls. In contrast, HCs with smaller proportions of uninsured patients were more likely to focus on improving coordination with external entities, record keeping, and HIT.

*Sustainability.* Few HCs described how solutions to improve CCS rates would be sustainable after the end of the funding period. However, examples of how these improvements may be sustained are illustrated by the following quotes.

*“The use of a RN Health Coach at [health center] will be sustainable by [health center] after the one year grant by utilizing the RN to the fullest extent of his/her credentials and relieving the provider to deliver an integrated care visit effectively and productively.”*

*“Additionally, we will be using this funding to hire care managers with consistent qualifications (Registered Nurse) to supplement our Family Support Worker (Care Coordinator) role, as well as to move the timeline up so we can provide comprehensive care management services, including pap recommendations, to high risk patients. We have identified future funding via local payment reform to make this a sustainable position.”*

### 3.2. Discussion

Our study found that HCs that have already attained PCMH recognition were more culturally sensitive in the preparation of educational materials and utilization of cultural brokers in patient navigation. Furthermore, these HCs have developed more flexibility in promoting access to screening through walk-in appointments and mobile clinics. The majority of HCs proposed multilevel approaches to improve CCS rates by simultaneously targeting patient-level, provider-level, and system-level barriers. Such multipronged approaches have been demonstrated to be more successful in serving low-income minority populations of underscreened women [28].

Overall, the most frequently proposed solution was at the system level, specifically enhancing utilization of HIT/EMRs (80% of subject HCs). EMR tools have been successful in assisting OB/GYN physicians in adherence to CCS guidelines transformation [29]. Since clinical data collection and use of data for population management in EMR systems are integral components of PCMH, it will be important to monitor its impact on improving the capacity of HCs primary care physicians to provide CCS and another preventive care [30]. Other common system-level solutions included changes in staffing such as either hiring new employees or increasing existing employees' hours as patient navigators, incorporating a quality improvement process, and implementing improved care management protocols [8]. At the provider level, over half of grantees proposed providing education or training for providers. The most frequently proposed patient-level solutions were to provide outreach, education, or health promotion for patients (74% of subject HCs) as well as to improve and/or increase patient communication (66% of subject HCs) [31]. The breadth of barriers and proposed solutions highlights the diversity of needs across HCs.

Patients may not receive up-to-date screening for a wide variety of reasons, including limited access to services due to geographic location or lack of insurance, psychological barriers such as mistrust of providers or fear of pain, or lack of knowledge regarding the need to schedule a preventive visit [32–34]. These attributes are reflective of the patient-level barriers described by the HCs with respect to cultural, health belief, and behavioral factors of the patients served through HCs. In a PCMH transformed practice, patients can maintain a continuous relationship with a team of health care providers, who are responsible for comprehensive, well-coordinated health care [35]. HCs utilizing the PCMH model may especially benefit the medically underserved, patients with specific conditions, and high-risk populations by alleviating health care access issues (e.g., through providing transportation or providing specialty services onsite) [36, 37]. In addition, HC patients have reported high quality of care in HCs with PCMH attributes related to access to care and communication [38].

*Limitations*. There are several limitations in this study. The project narratives data varied in writing quality and level of detail provided. Data gathering consisted of authors' review of project narratives by subject HCs. The authors did not interact with the subject HCs to further probe them for additional information. Furthermore, the coded data were based on subject HCs that may have been motivated to provide explanations of external factors for lower CCS rates. This may help to explain why so few HCs reported provider-level barriers to CCS. Finally, we did not conduct a probability sampling for 80 project narratives. Instead, we selected a purposive sample of 80 HCs, which had similar patient and institutional characteristics as the full cohort of HCs who received the funding.

## 4. Conclusions

This study sought to investigate barriers to CCS reported by HCs and proposed solutions to increase screening rates, among HCs undergoing PCMH transformation. Although PCMH transformation is associated with higher HC operating costs, three-quarters of all eligible HCs applied for PCMH supplemental funding, which is a strong indication of their eagerness to participate in PCMH transformation and improve quality of clinical care including increasing CCS rates [39]. During this PCMH transformation period, the USPSTF updated the CCS recommendation in 2012 for women over the age of 30 to receive a Pap test accompanied with an HPV test every five years as compared with every 3 years in the 2003 USPSTF recommendations [21, 40]. The HCs UDS clinical measures were subsequently revised in 2013 to align with the 2012 USPSTF CCS recommendations [41]. Future research is planned to continue the monitoring of CCS rates and the impact of PCMH transformation amidst changing clinical guidelines.

## Figures and Tables

**Table 1:** Comparison of all grantees and sample grantees by general health center characteristics and patient demographics.

	FY12 supplemental funding grantees (*n* = 809)	Final sample (*n* = 80)
*Health center characteristics *		
% large (>10,000 patients per year)	60.4%	57.5%
Mean clinical FTEs/10,000 patients	24.8	25.9
HHS regions		
% I	9.3%	8.8%
% II	9.3%	8.8%
% III	9.6%	8.8%
% IV	15.5%	15.0%
% V	13.8%	13.8%
% VI	10.9%	10.0%
% VII	5.3%	6.3%
% VIII	4.9%	5.0%
% IX	13.8%	15.0%
% X	7.5%	8.8%
% urban	52.1%	51.3%
Pap test quartile		
1 (highest 25% screening rate)	24.2%	21.3%
2	27.2%	26.3%
3	26.3%	28.8%
4 (lowest 25% screening rate)	22.1%	23.8%
% PCMH recognized	23.5%	23.8%
*Patient demographics *		
>10% homeless	6.7%	10.0%
>10% agricultural Worker	6.1%	7.5%
>10% uninsured	22.5%	23.8%
>10% Hispanic/Latino	33.9%	32.5%
>10% Asian	5.1%	6.3%
>10% African American	29.4%	26.3%
>10% best served in language other than English	17.4%	18.8%

FY: fiscal year. FTEs: full-time equivalents. HHS: Health and Human Services. PCMH: patient-centered medical home.

HHS regions: US Department of Health and Human Service divided the country into 10 regions with offices to oversee regional operations.

**Table 2:** Barriers to cervical cancer screening and solutions to increase screening rates.

Barriers		
Patient barriers	Provider barriers	System barriers
Demographic (*n* = 30)	Lack of HIT training (6)	Not using full capability of EMR (20)
Financial issues/uninsured (18)	Noncompliance with established protocols/guidelines (4)	Coordination with external labs or providers (13)
Transportation barriers (7)	Preventive care not embedded into practice (2)	Inadequate staff hours or employees: For patient education (3) For coordination (8)
Transient patient population (5)	General lack of education/training (2)	Limited clinical hours (3)
Lack of childcare (3)		No policy/procedure to document all practices (2)
High patient turnover rate (1)		Lack of supplies/equipment for procedures (2)
Other/general (13)		EMR not yet implemented (1)
Cultural factors (*n* = 20)		Lack accurate patient contact information (1)
Fear of procedure or results (9)		Health professional shortage area (1)
Language barrier (5)		No patient surveys (1)
Trust issues (4)		Primary care providers do not provide cervical cancer screening (1)
Discomfort with male providers (3)		No OB/GYN specialist at HC (1)
Immigration status (2)		
Health beliefs/behaviors (*n* = 21)		
Lack of awareness/knowledge of cervical cancer screening need (10)		
Not seeking preventive care (8)		
Do not keep appointments (7)		
Lack knowledge of available cervices (2)		
Late for appointments (1)		

	Solutions	
Patient-targeted	Provider-targeted	System-targeted

Outreach, education, or health promotion for patient population, 59 (74%)	Education/training for providers, 44 (55%)	Utilize HIT/EMR, 63 (79%)
Improve/increase communication with patients, 53 (66%)	Feedback to providers Team performance, 7 (9%) Individual performance, 3 (4%)	Staffing: hire new employee or increase hours, 46 (58%)
Facilitate access to care, 29 (36%)		Regular quality improvement process, 39 (49%)
		Implement protocol to better manage care, 32 (40%)
		Improve recording keeping, 24 (30%)
		Improve coordination with external providers, labs, 21 (25%)
